# Development of a Prototype Robotic System for Radiosurgery with Upper Hemispherical Workspace

**DOI:** 10.1155/2017/4264356

**Published:** 2017-07-24

**Authors:** Sun Young Noh, Kyungmin Jeong, Yong-chil Seo, Chang-hoi Kim, Jongwon Park, Yoo Rark Choi, Sung Uk Lee, Yeong-Geol Bae, Seungho Kim

**Affiliations:** Korea Atomic Energy Research Institute, Daejeon, Republic of Korea

## Abstract

This paper introduces a specialized robotic system under development for radiosurgery using a small-sized linear accelerator. The robotic system is a 5-DOF manipulator that can be installed above a patient to make an upper hemispherical workspace centered in a target point. In order to determine the optimal lengths of the link, we consider the requirements for the workspace of a linear accelerator for radiosurgery. A more suitable kinematic structure than conventional industrial manipulators is proposed, and the kinematic analysis is also provided. A graphic simulator is implemented and used for dynamic analysis. Based on those results, a prototype manipulator and its control system are under development.

## 1. Introduction

The aim of stereotactic radiosurgery systems such as Linac, Gamma Knife, and CyberKnife is to destroy tumor tissue while preserving adjacent normal tissue using ionizing radiation rather than excision with a blade [1, 2]. Thus, they should be able to accurately focus radiation beams from as many different angles as possible to converge on one target tumor [3, 4].

A Gamma Knife is used for treating not only brain tumors but also vascular and functional pathologies. And it typically contains 201 Co-60 radiation sources, each placed in a circular array, and emits gammy ray through a target point in the patient's brain. Thus, the Gamma Knife has over 200 beam delivery angles [5, 6].

Compared with the Gamma Knife, a Linac system uses X-rays generated from a linear accelerator, and the accelerator is mechanically rotated around the patient, in a full or partial circle to change the delivery angle aiming a target point. And the couch where the patient is lying can also be moved in small linear or angular steps. Thus, the combination of the movements of the gantry and of the couch can make more beam delivery angles. But they are confined within an approximately 2-dimensional space [7, 8]. A CyberKnife system also uses a linear accelerator as a radiation source, but relatively small and light-weighed. It uses a 6-DOF industrial robot manipulator to move the X-ray source to aim arbitrary points from different angles [9, 10]. So, CyberKnife systems are recognized as one of the most versatile radiosurgery systems having a broad range of the beam delivery angle in a 3-dimensional space [11, 12].

But even though the degree of freedom of the manipulator used in a CyberKnife system is 6, 5-DOF is sufficient to aim an arbitrary 3-dimensional point from an arbitrary delivery angle because the change in the rolling angle of the beam about its axis has no effects [13, 14].

And besides, whereas the required radius of workspace for the radiosurgery is about 1 meter and the maximum moving speed of the end-effector is less than 1 m/s, the maximum radius of the workspace in the CyberKnife system is approximately 3 meters and its moving speed is also higher than required. That means that the performance of the joint-driving motors of industrial robot manipulators in the market is usually higher than required, and the driving motors are oversized. It may cause the system to be heavy-weighted and make its cost high. Furthermore, in cases when the robot manipulator is installed on the floor beside the couch, there are limitations for increasing the range of the beam delivery angle.

So, this paper introduces a specialized robotic system under development for radiosurgery using a small-sized linear accelerator. The robotic system is a 5-DOF manipulator installed above a patient to make an upper hemispherical workspace centered in a target point.

In Section 2, the basic structure and the design process for determining the lengths of its links are described.  Section 3 provides the kinematic analysis of the manipulator, and Section 4 explains a graphic simulation for dynamic analysis and its results. The manufactured manipulator under development and the structure of control system are shown in Section 5.

## 2. Design of Lengths of Robot Arm Links

As mentioned above section, the manipulator for radiosurgery needs 5-DOF joints in minimum to deliver a linear accelerator, so the lengths of the links should be optimized for its operation. And also, the manipulator workspace takes into consideration the position of the patient and is designed to avoid contact with the patient. This is achieved by creation of a safety zone around the patient and the treatment couch.

**Figure 1:**
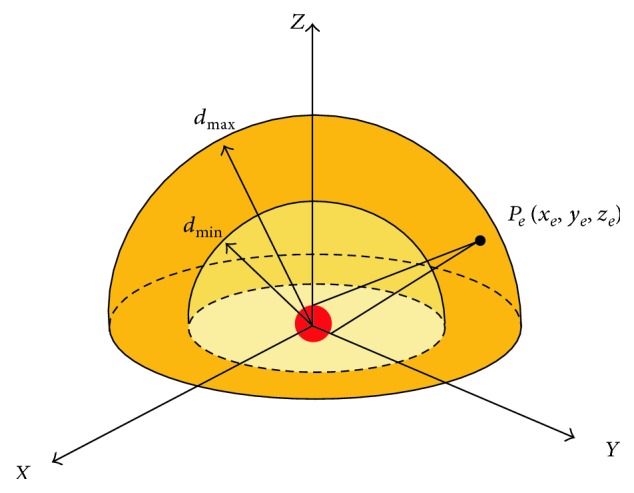
Workspace for radiosurgery.

In Figure 1, the center of a tumor tissue to be treated is assumed to be located at the origin of xyz coordinates. The *x*- and *y*-axes define a horizontal plane, and the *z*-axis is vertical.

Let (*x*_*e*_, *y*_*e*_, *z*_*e*_) refer to the position of X-ray source inside the linear accelerator.

If SAD (source-to-axis distance) is between *d*_min_ and *d*_max_, and the workspace is limited to the upper hemisphere, then the required workspace where the end point should reach at is

(1) $$\begin{array}{c} d_{\min}\leq \sqrt{{x_{e}}^{2}+{y_{e}}^{2}+{z_{e}}^{2}}\leq d_{\max},\\ z_{e}\geq 0. \end{array}$$

At any point in the workspace, the accelerator should direct radiation beams to a small region around the origin.

**Figure 2:**
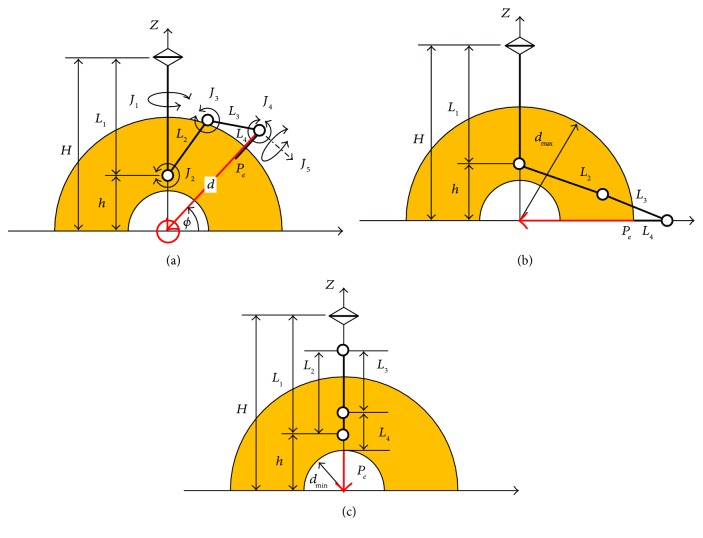
Proposed configuration of robot.

In our study, a serial manipulator with revolute joints is considered as shown in Figure 2(a).

The joint 1 (*J*_1_) is aligned with the *z*-axis and defines a central sagittal plane, and by using joints 2 (*J*_2_), 3 (*J*_3_), and 4 (*J*_4_) parallel to each other, the accelerator is directed to the origin at any point in that plane. Both joints 4 and 5 (*J*_5_) are used to direct the beam to the neighboring region.

The center of joint 2 is shifted above the origin with *h* to avoid collision with a patient.

One extreme constraint with the lengths of the links is the case when *ϕ* = −*π*/2, *d* = *d*_max_ where the angle *ϕ* is defined as shown in Figure 2(a), and the reach of the arm is maximum as shown in Figure 2(b). To access such a posture in its full reach, the lengths of the links satisfy the following equation:

(2) $$\begin{array}{c} L_{2}+L_{3}=\sqrt{h^{2}+{\left(d_{\max}+L_{4}\right)}^{2}}. \end{array}$$

Another case is when *ϕ* = *π*/2, *d* = *d*_min_ → *ϕ* = −*π*, *d* = *d*_min_. In that case, the following equation satisfies the posture:

(3) $$\begin{array}{c} h+L_{2}-L_{3}-L_{4}=d_{\min}. \end{array}$$

The link's lengths *L*_2_, *L*_3_, *L*_4_ satisfy (2) and (3), and the required workspace of the arm is obtained.

When *d*_min_, *d*_max_, *L*_4_ are given, *L*_2_ and *L*_3_ can be determined as follows:

(4) $$\begin{array}{c} L_{2}=\frac{1}{2}\left(\sqrt{h^{2}+{\left(d_{\max}+L_{4}\right)}^{2}}-h+L_{4}+d_{\min}\right),\\ L_{3}=\frac{1}{2}\left(\sqrt{h^{2}+{\left(d_{\max}+L_{4}\right)}^{2}}+h-L_{4}-d_{\min}\right). \end{array}$$

Table 1 shows *L*_2_ and *L*_3_ determined from (4) when *d*_min_ = 0.6*m*, *d*_max_ = 1.0*m*, *L*_4_ = 0.1*m* optimal length according to *H*.

## 3. Kinematic Analysis

This section provides analysis for forward and inversed kinematics of the radiosurgery manipulator based on the proposed structure and the lengths of links.

### 3.1. Forward Kinematic Analysis

**Figure 3:**
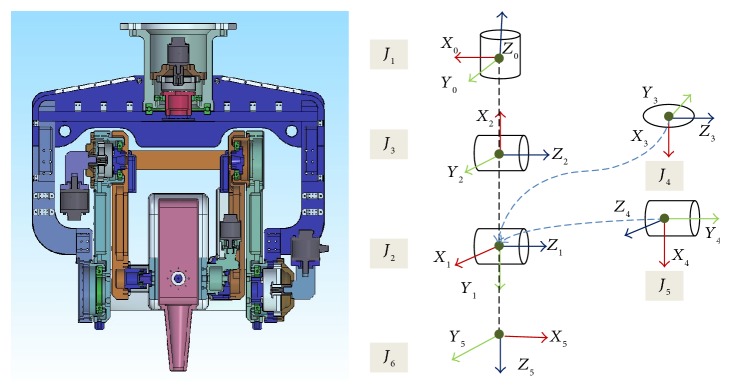
D-H parameters layout for manipulator.

Figure 3 shows the outline of the manipulator and its coordinates in D-H convention. The base coordinate system is located on the mounting plate of the manipulator, and the origin of the coordinate systems 3 and 4 is coincident with each other. The joints 2 and 3 are supported by two links in both sides to reduce deflections. And the dummy joint 6 is not really actuated but added to follow conventional kinematic analysis procedure for 6-DOF manipulators.

Table 2 provides the D-H parameters in mm for length and in degree for angle.

Using the D-H parameters, homogeneous transform matrices are given by

(5) $$\begin{array}{c} {}^{i}T_{i-1}=\left[\begin{array}{cccc} {\cos\theta }_{i} & -{\sin\theta }_{i} & 0 & a_{\left(i-1\right)}\\ {\sin\theta }_{i}{\cos\alpha }_{\left(i-1\right)} & {\cos\theta }_{i}{\cos\alpha }_{\left(i-1\right)} & -{\sin\alpha }_{\left(i-1\right)} & -{\sin\alpha }_{\left(i-1\right)}d_{i}\\ {\sin\theta }_{i}{\sin\alpha }_{\left(i-1\right)} & {\cos\theta }_{i}{\sin\alpha }_{\left(i-1\right)} & {\cos\alpha }_{\left(i-1\right)} & {\cos\alpha }_{\left(i-1\right)}d_{i}\\ 0 & 0 & 0 & 1 \end{array}\right]. \end{array}$$

By multiplying all homogeneous transform matrices sequentially from the left to the right, the final homogeneous matrix is obtained as follows:

(6) T10T21T32T43T54T65=T60=nxoxaxPxnyoyayPynzozazPx.

### 3.2. Inverse Kinematic Analysis

To calculate joint angles from a given target position and orientation, the central position of the accelerator, the origin of the coordinate system 3 is derived at first by the following equation:

(7) $$\begin{array}{c} P_{m}={\left[P_{x}\;P_{y}\;P_{z}\right]}^{\prime }-L_{6}\times W\left(L_{6}=\left\|\theta_{6}\right\|\right). \end{array}$$

Like a usually 6-DOF manipulator, there are 4 sets of solution for *θ*_1_~*θ*_3_ in maximum depending to its configurations such as the left or the right arm, elbow up or down. There are two sets of *θ*_4_ and *θ*_5_ that are determined to satisfy the given orientation for each set of *θ*_1_~*θ*_3_.

Thus, 8 sets of solution can be found if the given end point is inside the workspace. Among them, the joint angle limit criteria are applied to screen the solutions and the solution that is nearest from the current joint configuration is selected as the final solution.

A high continuous path can make smooth and efficient movements. To improve the continuity of the path, two successive points are interpolated with spline curves that consist of 5th-order polynomial equation and satisfy given points, velocities, and accelerations as follows:

(8) $$\begin{array}{c} S\left(t\right)=a+bt+{ct}^{2}+{dt}^{3}+{et}^{4}+{ft}^{5}. \end{array}$$

## 4. Graphic Simulator and Dynamic Analysis

In this study, a MATLAB-based open source code, ARTE (A Robotics Toolbox for Education) [15], is used and modified to simulate the motion of the radiosurgery manipulator under development in 3-dimensional graphic virtual environments.

To input geometric model of the manipulator to the simulator, 3D CAD models are converted to STL files in ASCII format.

And such dynamic parameters as masses, mass moments of inertia, and centers of masses for each link are also extracted from the 3D CAD files to enable dynamic simulation capability implemented in the toolbox.

**Figure 4:**
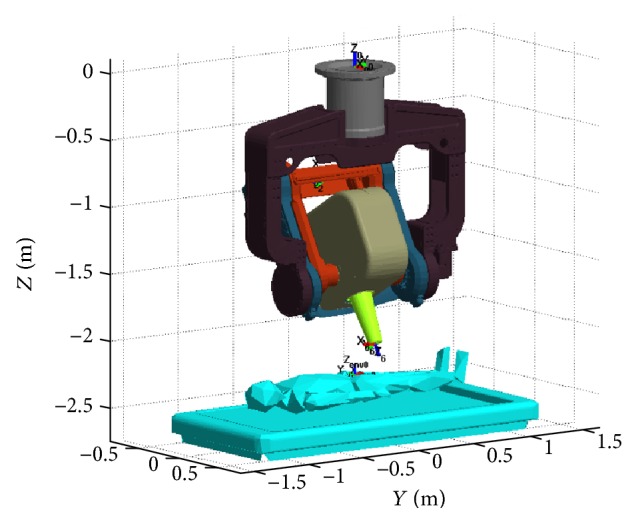
The 3D graphic simulation environment.

Figure 4 shows the manipulator and a patient lying on a couch in the 3D graphic simulation environment.

**Figure 5:**
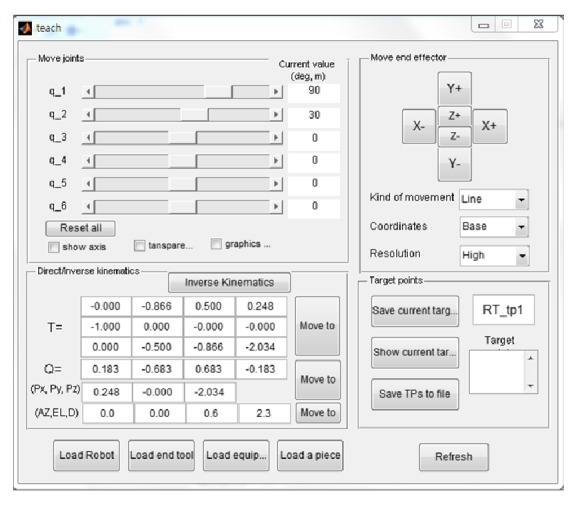
The control panel window.

Figure 5 shows the control panel window that displays current joint angles and target inputs to move the manipulator. It is modified from the teach program packaged in ARTE.

To command the manipulator to move, the azimuth angle, the elevation angle, and the distant from the origin are directed by operators. The desired target postures can be saved to text files for later dynamic simulations.

**Figure 6:**
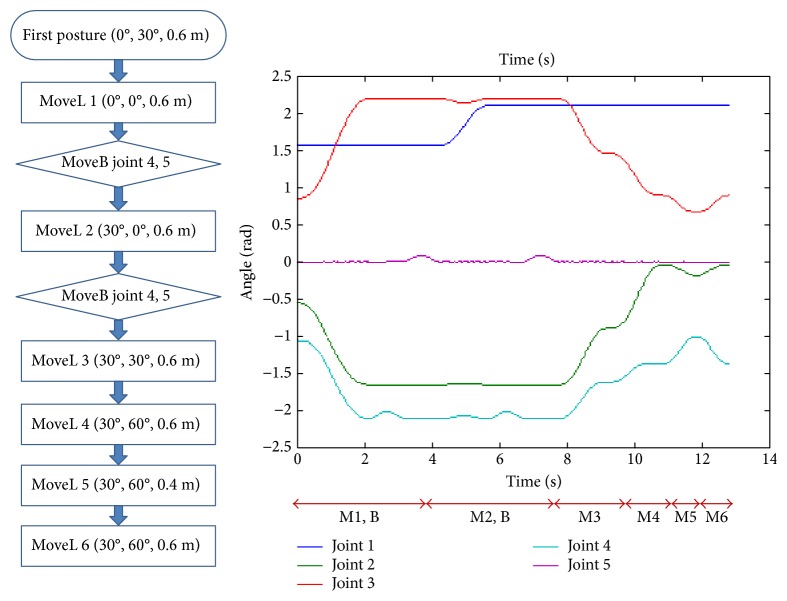
An example trajectory six postures.

As an example of trajectory, six postures are given as shown in Figure 6() using a function named MoveL() and the joints 4 and 5 are rotated slightly about 5^0^ in some posture using a function named MoveB() that independently moves the joints without moving all the other joints.

**Figure 7:**
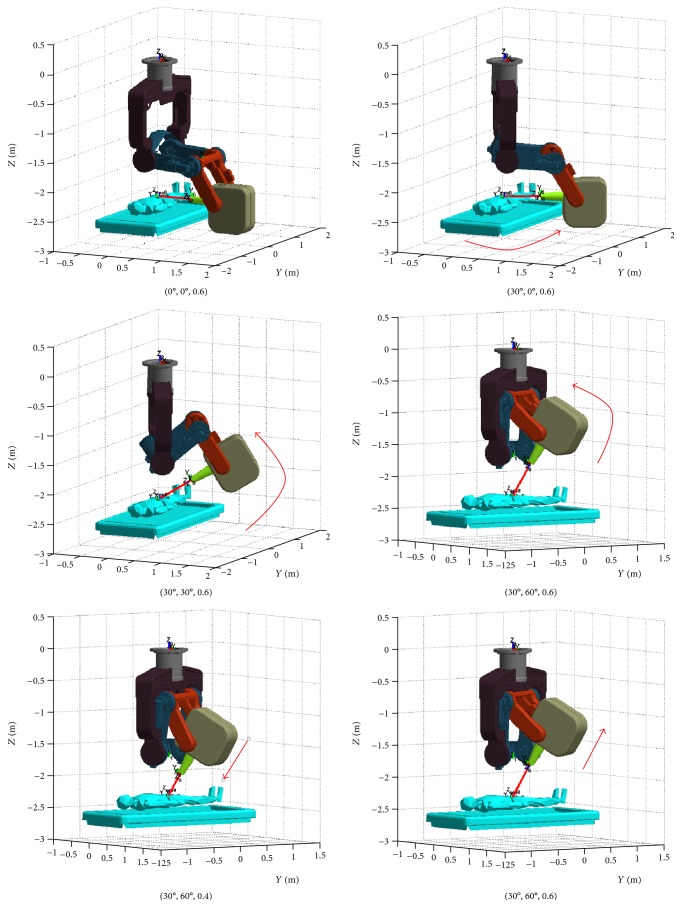
The six postures in the 3D graphic environments.

**Figure 8:**
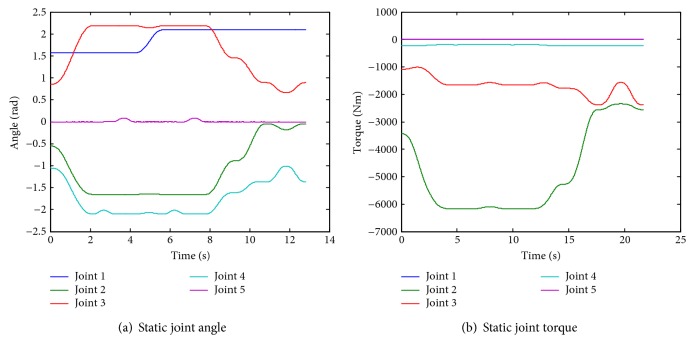
Joint angle and static torque.

Figure 7 shows the six postures in the 3D graphic environments, and Figure 8 shows the static joint torques.

**Figure 9:**
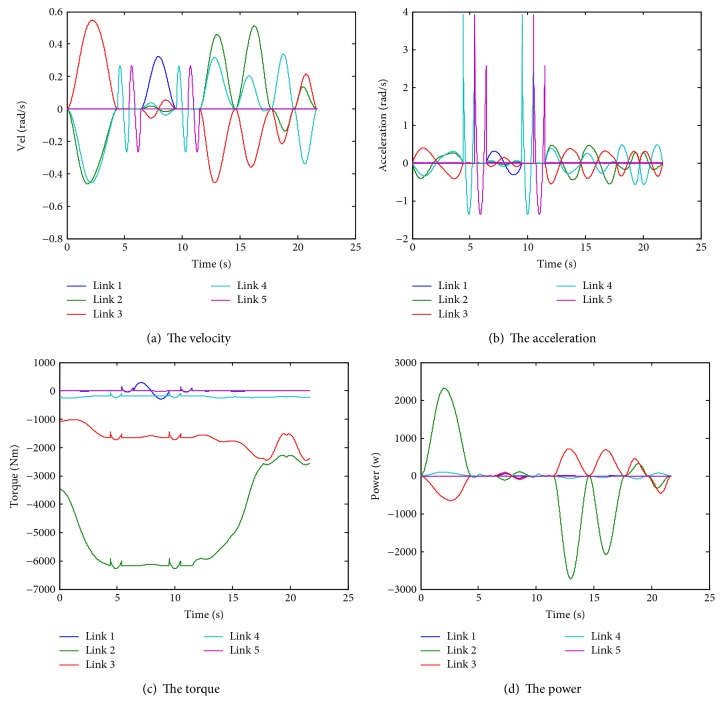
Simulation results.

As a dynamic simulation, the linear velocity of the end point is given 0.1 m/s, and the velocity, the acceleration, the torque, and the power of each joint are provided in Figure 9.

**Figure 10:**
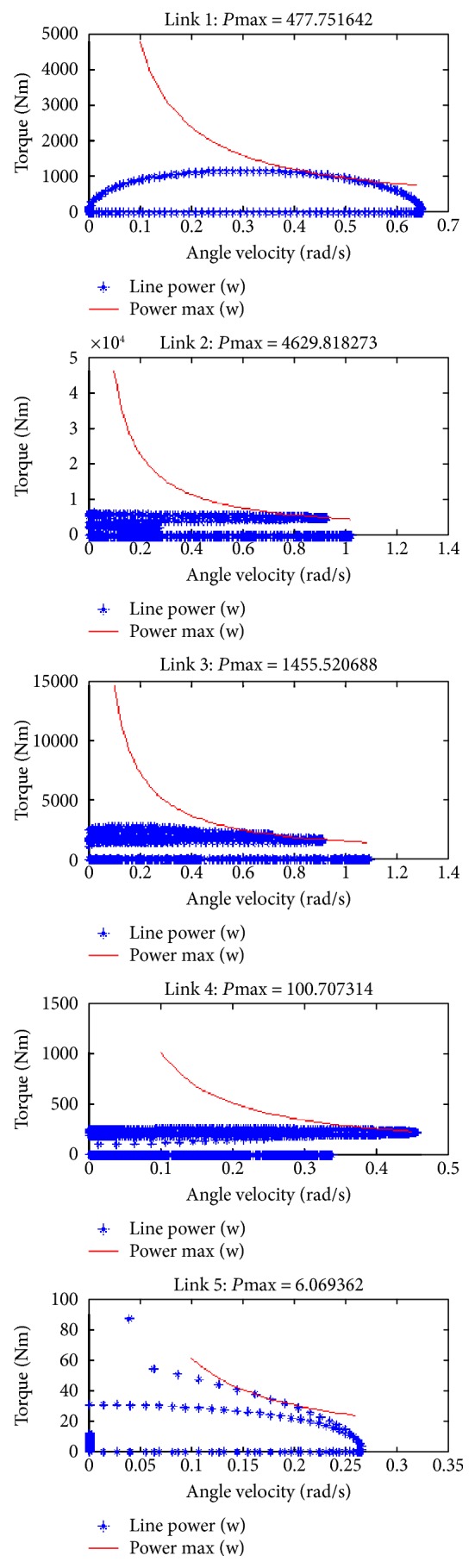
S velocity-torque diagram.

Figure 10 shows the joint velocity, the joint torque, and the power in velocity-torque diagram for each joint to help motor selection for performing the simulated motion.

As shown in Table 3, the maximum torque and power for the joint 2 is 6271 Nm and 2317 W.

## 5. Manufactured Robot and Control System

**Figure 11:**
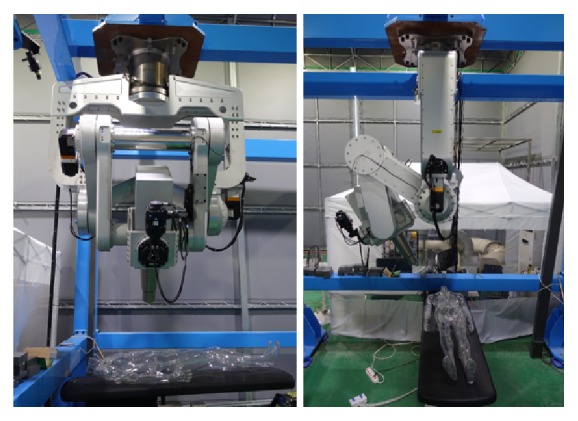
Prototype manipulator.

The motors and gearboxes are selected based on the dynamic simulation results, and the manipulator has been manufactured as shown in Figure 11.

As for controlling motors, MR-J4-B series servo amplifiers manufactured by Mitsubishi electric company are selected. They have an interface to a battery-backup optical encoder, and so there is no need to start initialization process at every power-up stage.

Those of 5 servo amplifiers are linked through SSCNET optical fibers to MR-MC 241 multiaxis position control board mounted in a PCI slot of a desktop computer operated in MS Windows 7. And also, we consider two types of safety features of the robotic system when danger exists on machines and systems they have to be immediately shut down, in order to protect people, machines, and systems. At first, stoppers are attached to the side of the joint 2 of the manipulator. It can be sure to take preventive method to the manipulator against falling to patients when danger exists on machines. Second, an emergency stop button and a signal emergency stop button in control system are designed to happen only in emergency situations. Pressing the emergency stop button, brakes, is activated in the manipulator axis, and the robot stops. The motor power turns off at this timing.

**Figure 12:**
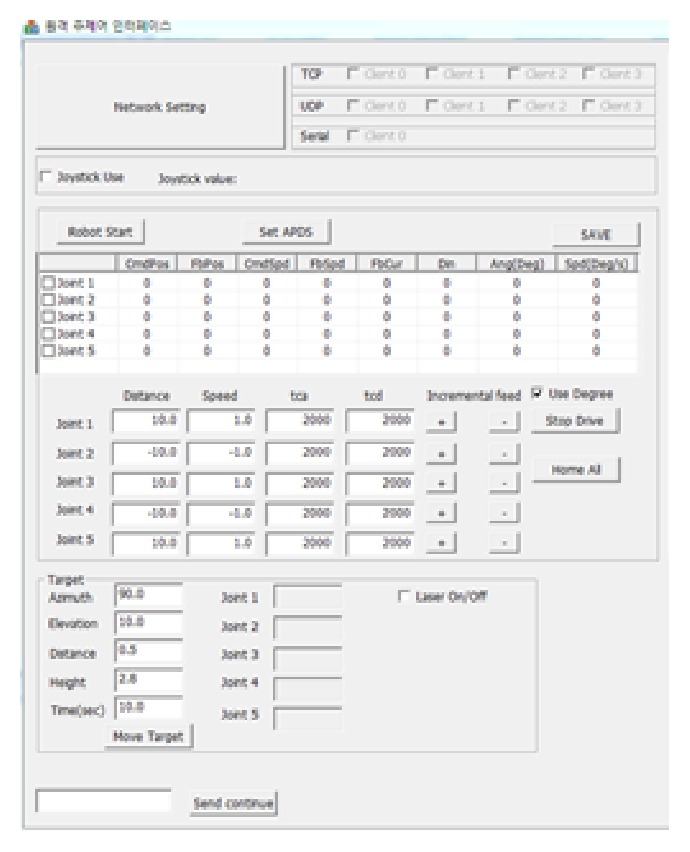
Graphical use interface.

Figure 12 shows the test GUI programed in MS Visual C++ environment. This program has included several functions implemented in the simulator.

## 6. Conclusion

This paper introduces a radiosurgery manipulator system that has 5-DOF and a more suitable workspace for its operation. This is achieved by creation of a safety zone around the patient and the treatment couch. In order to design the manipulator, kinematic and dynamic analyses are conducted and implemented in a 3D graphic simulator based on one of MATLAB-based open source robotics toolboxes, ARTE. Resulted from some motion simulations for the manipulator, a prototype manipulator is manufactured, and its control system is implemented.

## Figures and Tables

**Table 1:** Optimal length.

*H*	*L*_2_	*L*_3_
0.5	0.8	0.5
0.6	0.78	0.58
0.7	0.75	0.65
**0.8**	**0.73**	**0.73**
0.9	0.7	0.8
1.0	0.69	0.89
1.1	.67	0.97

**Table 2:** D-H parameters.

Joint number (*i*)	*a*_*i*-1_ (m)	∝_*i*-1_ [deg]	d_*i*_ [m]	*θ*_*i*_ [deg]
1	0	−90	−1.604	90
2	0.8	0	0	−90
3	0.73	0	0	180
4	0	90	0	0
5	0	−90	0	90
6	0	180	−0.567	0

**Table 3:** Maximum torque and power.

	Joint 1	Joint 2	Joint 3	Joint 4	Joint 5
Max. torque	289.61	6271	2446	250.60	87.17
Max. power	59.41	**2318**	718.32	100.7	6.06
